# Editorial: The mechanisms of parturition and preterm birth

**DOI:** 10.3389/fendo.2023.1179856

**Published:** 2023-04-04

**Authors:** Junjie Bao, Robert E. Garfield

**Affiliations:** ^1^ Guangzhou Key Laboratory of Maternal-Fetal Medicine, Guangzhou Women & Children’s Medical Center, Guangzhou Medical University, Guangzhou, China; ^2^ Department of Obstetrics and Gynecology, The University of Arizona College of Medicine-Phoenix, Phoenix, AZ, United States

**Keywords:** preterm birth (PTB), Electromyography (EMG), electrohysterogram (EHG), predict, parturition, biomarker

Parturition is a complex process involving changes at the hormonal, physiological, morphological, immune, and metabolic levels. Worldwide, preterm birth (PTB), defined as parturition occurring between 20 and 37 weeks of gestation, is a major determinant of neonatal mortality and morbidity, often with long-term adverse effects on the health of both mother and child.

In recent decades, the incidence of PTB has continued to rise in most countries. According to the World Health Organization, the rate of PTB across 184 countries is reported to be 5~18% of all births. Therefore, it is urgent to find ways to prevent preterm labor, and for this, a better understanding of the mechanisms behind it and those associated with parturition is necessary.

In this Research Topic we bring together important papers that carefully disclose the mechanisms of parturition and preterm birth and provide potential technologies and biomarkers to understand and predict them. This will definitely help us find more effective clinical interventions to prevent preterm birth and improve the outcome of parturition in the future.

Innovation of instrumentation and methods remains the major step in an understanding of the processes involved in birth. The use of electromyography (EMG) for parturition and preterm birth prediction has been found to have higher specificity and sensitivity (1, 2). Recently, H Liu’s group graded uterine EMG activities during labor and suggested Low-grade EMG indicated high possibility of oxytocin treatment in the follow-up labor process and also longer first stage and total labor time (3). In this Research Topic, Romero-Morales et al. enhanced classification of preterm-term birth using continuous wavelet transform and entropy-based methods of electrohysterogram (EHG) signals, and Qi et al. reported two fetal membrane indicators derived from Magnetic Resonance Imaging that can predict premature rupture of membranes (PROM) and preterm premature rupture of membranes (PPROM). Olmos-Ramírez et al. have also found that uterine activity modified responses of the fetal autonomic nervous system at preterm active labor using a novel time-frequency analysis of fetal heart rate variability (FHRV). Besides this significant progress, Hussein et al. reported lowered expression of soluble Fms-like tyrosine kinase 1 (sFLT-1) and placental growth factor (PlGF) with high ratio of sFLT-1/PlGF in extracellular vesicles of preterm birth and suggested that could be used as potential biomarkers for preterm birth.

With the progress of technologies, H Liu’s group also published a series of papers in Frontiers using transcriptome sequencing (RNA-seq), mass spectrometry detection of proteome (LC-MS/MS), single cell transcription sequencing (scRNA-seq), and spatial transcription (ST). In addition, they verified the involvement of inflammation and hypoxia in laboring myometrium and that gives some important hints for future studies (4–6).

In this Research Topic, Vidal et al. submitted a significant review about the multiple feto-maternal organ systems, differing mechanisms, and pathways involved in spontaneous preterm birth. They also stressed the need for biological models that permit concomitant observation and analysis of organ interactions, rather than focusing on these tissues as single entities.

In summary, the papers in this Research Topic help us to better understand the mechanisms of parturition and preterm birth and shed new light into more effective clinical predictions and interventions in the future.

## Author contributions

JB wrote the first draft of the manuscript, and RG revised the manuscript. All authors read and approved the final version.
